# Supplementary material for: Irrational choices via a curvilinear representational geometry for value
Source: Nat Commun. 2024 Jul 30;15:6424. doi: 10.1038/s41467-024-49568-4 (PMC11289086; doi:10.1038/s41467-024-49568-4)
Supplement: Supplementary file 1 — Supplementary Information [file 41467_2024_49568_MOESM1_ESM.pdf]

Supplementary Information

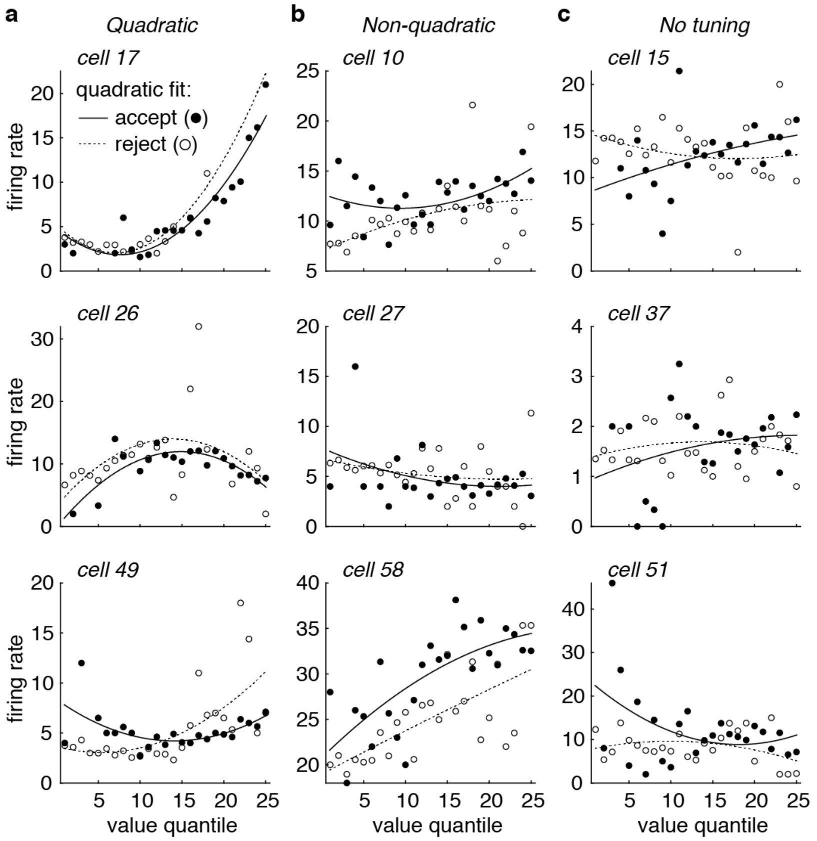

Figure S1. Tuning for value within accepted and rejected offers in individual vmPFC neurons, related to Figure 2. *The firing rates of example neurons presented in Figure 1, plotted separately for accepted (filled circles) and rejected (open circles) offers as a function of the original value quantile bins.* ***A)*** *The firing rates of three example neurons (rows) that were quadratically tuned for value.* ***B)*** *Same as (A) for three example non-quadratically tuned neurons.* ***C)*** *Same as (A) for three example neurons that were not tuned for value. The lines show best quadratic fit to the accepted (solid line) and rejected (dotted line) offers.*

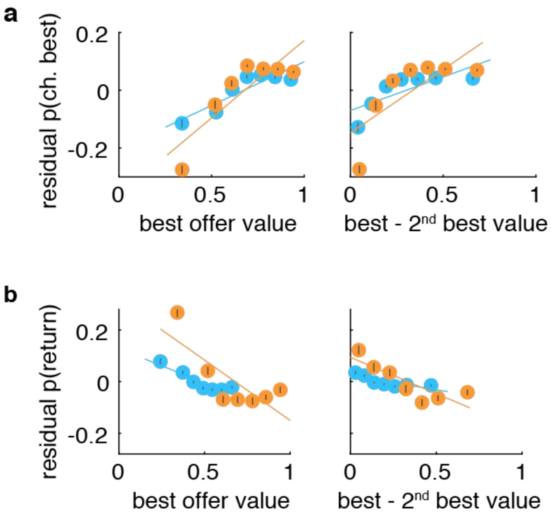

Figure S2. Effects of best offer and best-2nd best offer values on residual behavioral performance, related to Figure 1. A) *Probability of choosing the best option as a function of best offer value after regressing out best-2nd best offer value (left, both subjects: 2.84, 95% CI = [2.65, 3.03], t(85) = 29.66, p < 0.001, subject J: 2.40, 95% CI = [2.20, 2.60], t(44) = 24.04; subject T: 3.32, 95% CI = [3.05, 3.59], t(40) = 24.92, both p < 0.001), and as a function of best-2nd best offer value after regressing out best offer value (right, both subjects: 2.64, 95% CI = [2.48, 2.80], t(85) = 32.33, p < 0.001, subject J: 2.31, 95% CI = [2.11, 2.51], t(44) = 23.41, subject T: 3.00, 95% CI = [3.05, 3.59], t(40) = 24.92, both p < 0.001), multiple offer trials.* ***B)*** *The same as (A) but for returning to a previously seen option (returning to a previous option as a function of best offer value after regressing out best-2nd best offer value, both subjects: -2.27, 95% CI = [-2.49, -2.04], t(85) = -20.11, p < 0.001, subject J: -2.08, 95% CI = [-2.39, -1.78], t(44) = -13.73; subject T: -2.47, 95% CI = [-2.81, -2.14], t(40) = -15.07, both p < 0.001); returning to a previous option as a function of best-2nd best offer value after regressing out best offer value, both subjects: -1.28, 95% CI = [-1.45, -1.12], t(85) = -15.21, p < 0.001, subject J: -1.38, 95% CI = [-1.64, -1.13], t(44) = -10.90; subject T: -1.18, 95% CI = [-1.39, -0.96], t(40) = -10.82, both p < 0.001). Lines = least squares fit. Error bars indicate +- standard error of the mean across sessions (SEM), subject J: n sessions = 45, subject T: n sessions = 41.*

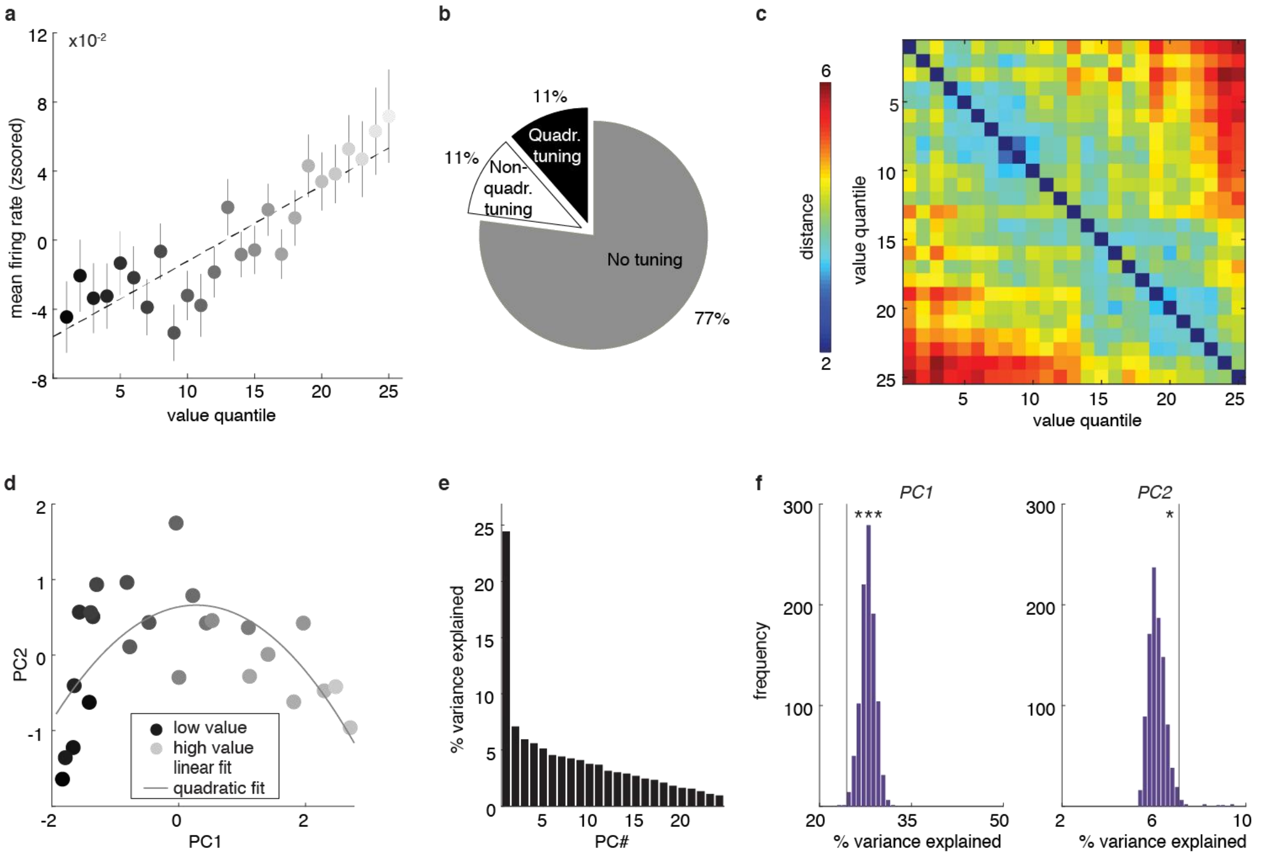

Figure S3. Representational geometry of value 100-300 ms after presentation of the offer, rather than the* a priori *epoch, related to Figure 2 and 3. A) *Average firing rates from all 122 neurons, plotted as a function of value quantile. Error bars indicate +- standard error of the mean across neurons (SEM).* ***B)*** *Proportion of all cells (n = 122) that were quadratically tuned, non-quadratically tuned or had no tuning for value.* ***C)*** *The mean distance between neuronal states corresponding to different values.* ***D)*** *The projection of the neural population onto the first 2 principal components (PCs). Shades of gray = value bins from low (light gray) to high (dark gray). Dotted line = best linear fit. Solid line = best quadratic fit.* ***E)*** *Percent variance explained by each PC.* ***F)*** *A comparison of the variance explained by the first 2 PCs in the real population (vertical line) against bootstrapped distributions of linearized datasets.***p = 0.001; *p = 0.024.*

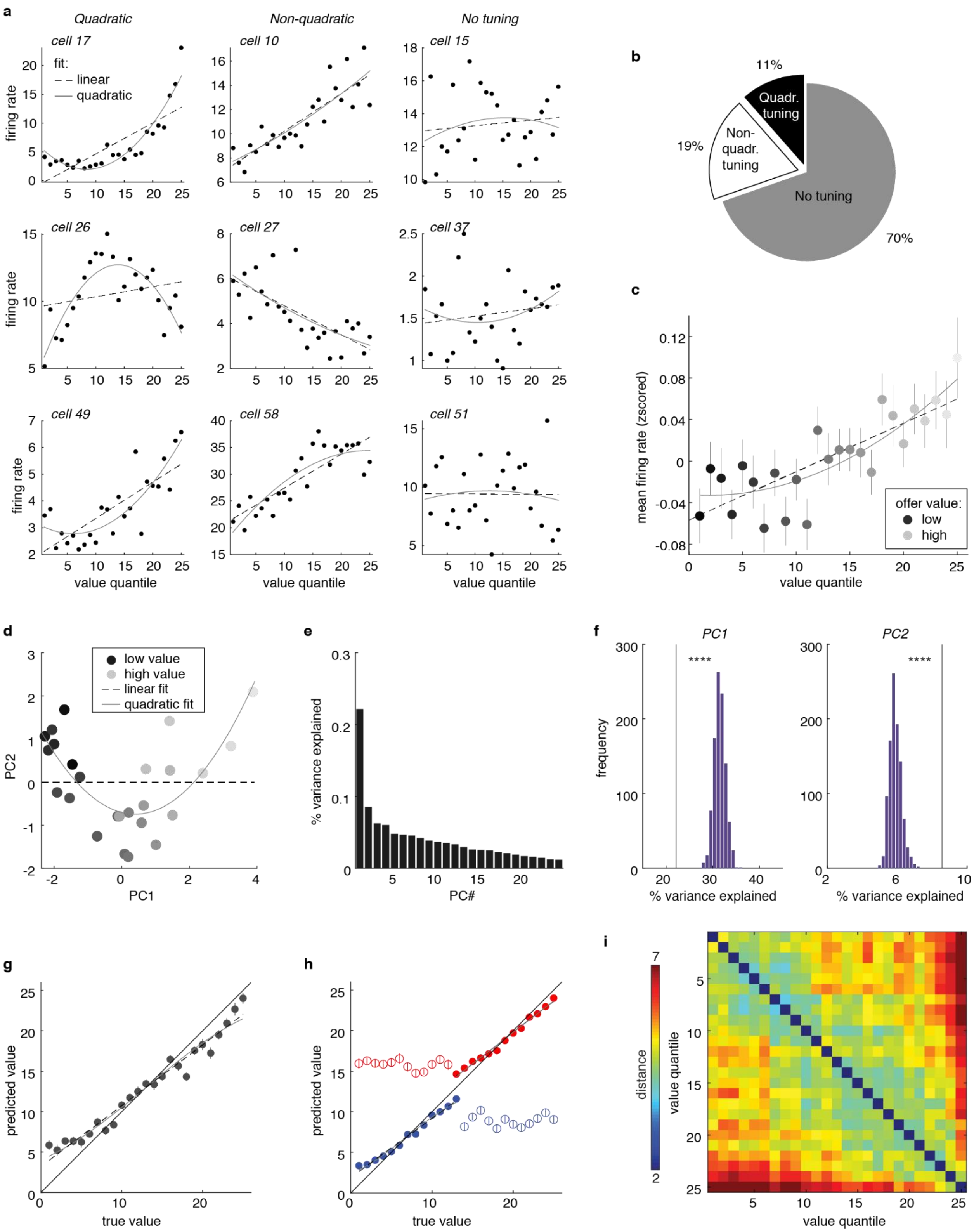

Figure S4. Representational geometry of value based on first offers revealed, related to Figure 2, 3 and 4. A) *The firing rates of example neurons that were quadratically (left column) or non-quadratically (middle column) tuned for value or not tuned for value (right column),*

plotted as a function of value quantile bins. ***B)*** *Proportion of all cells (n = 122) within each category.* ***C)*** *Average firing rates from all 122 neurons, plotted as a function of value quantile. Error bars indicate +- standard error of the mean across neurons (SEM).* ***D)*** *The projection of the neural population onto the first 2 principal components (PCs). Shades of gray = value bins from low (light gray) to high (dark gray). Dotted line = best linear fit. Solid line = best quadratic fit.* ***E)*** *Percent variance explained by each PC.* ***F)*** *A comparison of the variance explained by the first 2 PCs in the real population (vertical line) against bootstrapped distributions of linearized datasets.* ***G)*** *A linear decoder trained on the vmPFC population and used to predict value.* ***H)*** *Decoders trained on the population response to one half of the values (filled circles) and used to predict values outside of this range (open circles). Red = trained on high values; blue = trained on low values.* ***I)*** *The mean distance between neuronal states corresponding to different values. Error bars indicate +- standard error of the mean across neurons (SEM), n = 122. ****p < 0.001.*

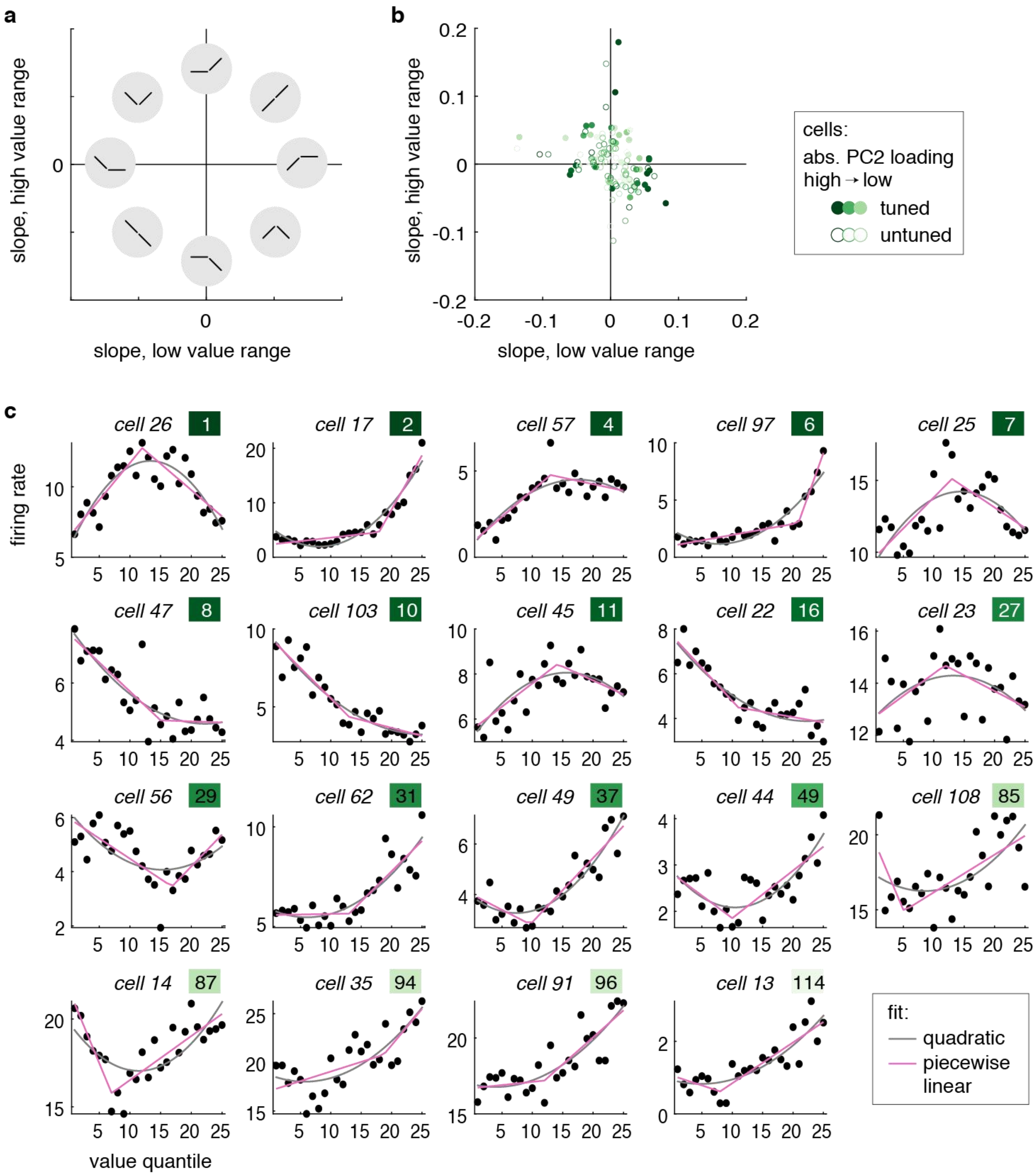

Figure S5. Piecewise linear regression of firing rates depending on offer value, related to Figure 2. A) *Schematic representation of slope orientations in piecewise linear regression.* ***B)*** *Each point represents one neuron that was either significantly tuned (filled circles) or untuned (open circles) to the offer value according to the test based on mutual information between firing rates and value labels (see* ***Methods****). Shading indicates absolute PC2 loadings, with the darkest green for cells with the highest contributions (see* ***Supplementary Figure S6****). The piecewise linear model fits independent slopes to neural responses to high and low values, with the break point constrained to fall between the 20% lowest and the 20% highest values. Points along the vertical axis only have a slope for high values, points along the horizontal axis only have a slope for low values. There was an overall negative correlation between lower and higher value slopes (r = -0.28, p = 0.002), supporting the finding of non-linear shapes in single*

cell responses. Less than 20% [9/46] of the tuned neurons were better described with piecewise linear fit than the curvilinear fit (Mandel's test, see ***Methods****) suggesting that floor (downward rectified) or ceiling (upwards rectified) effects were not a better explanation for the majority of the tuned responses. Quadratic fit was better than piecewise linear fit in ~13% [6/46] of tuned cells, leaving ~67% [31/46] of them not better described by either piecewise linear or quadratic fit.* ***C)*** *The firing rates of all non-linearly tuned neurons, plotted with quadratic (gray line) and piecewise linear (pink line) fits. The green boxes with a number indicate the cell's rank along PC2 absolute loadings (see* ***Supplementary Figure S6****).*

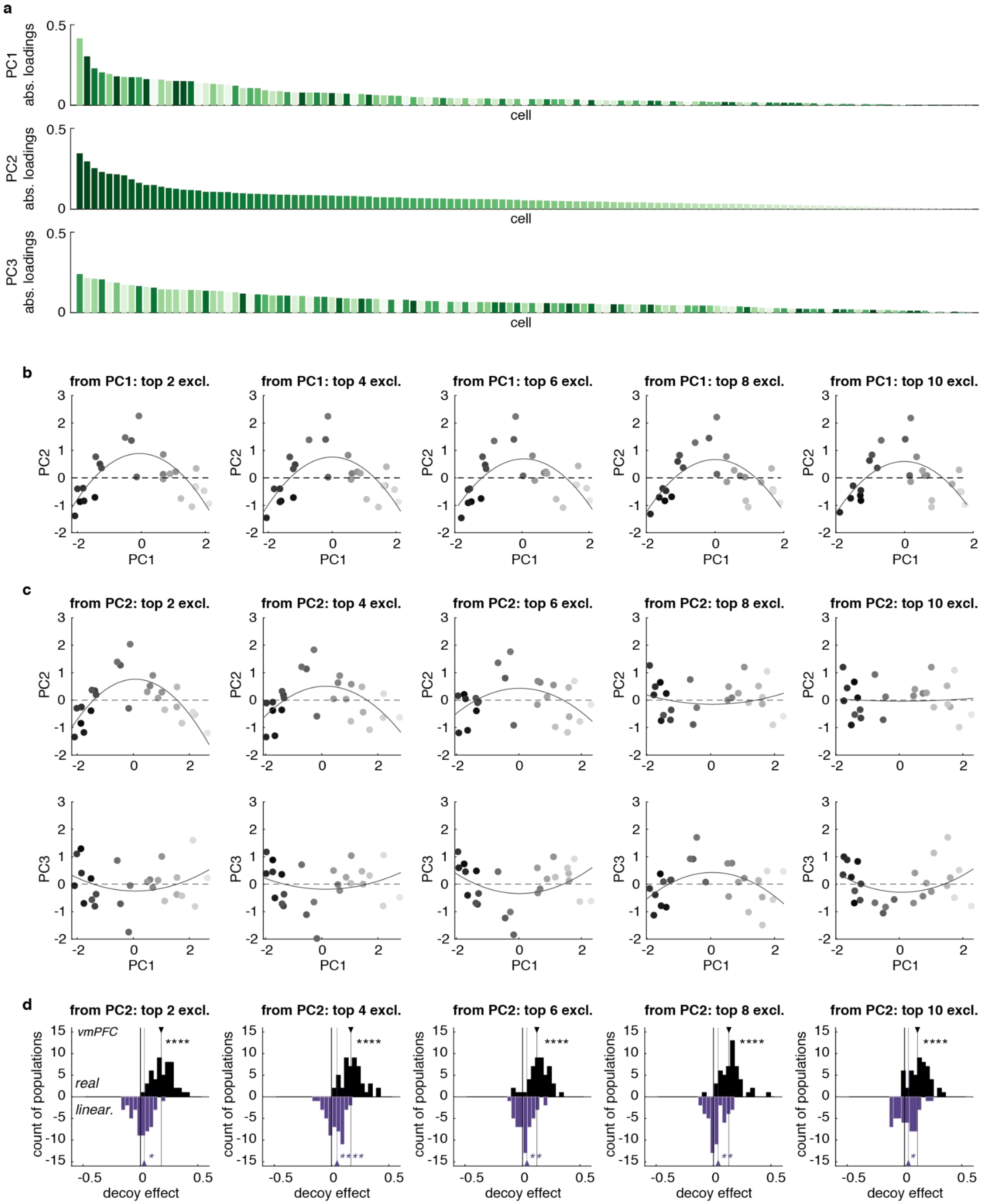

Figure S6. Contribution from single cells to the population manifold, related to Figure 3 and Figure 5. A) *PC loadings for PC1, PC2 and PC3 across the neurons sorted from highest to lowest. Cells are color-coded by their rank on PC2.* ***B)*** *The projection of the neural population onto the first 2 principal components (PCs) after excluding 2, 4, 6, 8 or 10 of the neurons with*

highest weights in the PC1. Excluding the neurons with highest PC1 loadings has little impact on the visible curvature of the projection of the manifold. ***C)*** *The projection of the neural population onto PC1 and PC2 or PC1 and PC3 after excluding 2, 4, 6, 8 or 10 of the neurons with highest weights in the PC2 (see also* ***Supplementary Figure S5*** *for piecewise linear regression weights for these neurons). Excluding the neurons with highest PC2 loadings removes the curvature from the PC1 vs PC2 projection. However, curvature can still be found in PC3. Shades of gray = value bins from low (light gray) to high (dark gray). Dotted line = best linear fit. Solid line = best quadratic fit.* ***D)*** *Distributions of decoy effect slopes from vmPFC pseudopopulations after excluding 2, 4, 6, 8 or 10 of the neurons with highest weights in the PC2 (black) and their linearized version (purple). Filled arrows = means of the distribution. *p < 0.01, **p < 0.001, ****p < 0.001.*

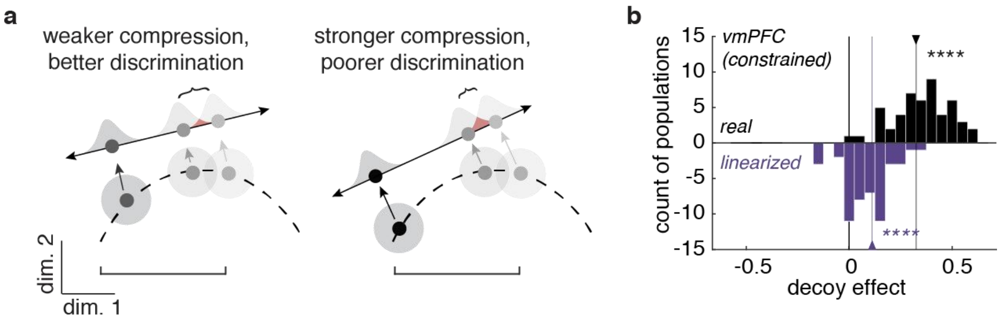

Figure S7. Decoding from a constrained range of values in vmPFC (real and linearized), related to Figure 5. A) *A cartoon illustrating how the ability to discriminate good offers could depend on how bad the worst offer is in a curved manifold, when the best values in the set are around the middle of the full range of the manifold and the decoding range is thus constrained (black horizontal braces).* ***B)*** *Distributions of decoy effects from vmPFC pseudopopulations (black) and their linearized version (purple) value manifold constrained to the lower 56% of values (value bins 1-14 from the full range of 25, see* ***Methods****, vmPFC population, mean decoy effect slope = 0.326, 95% CI = [0.286, 0.366], t(49) = 16.37, p < 0.001, one-sample t-test from 0; linearized population, mean decoy effect slope = 0.111, 95% CI = [0.081, 0.141], t(49) = 7.40, p < 0.001, one-sample t-test from 0). Filled arrows indicate the means of the distributions. ****p < 0.001.*

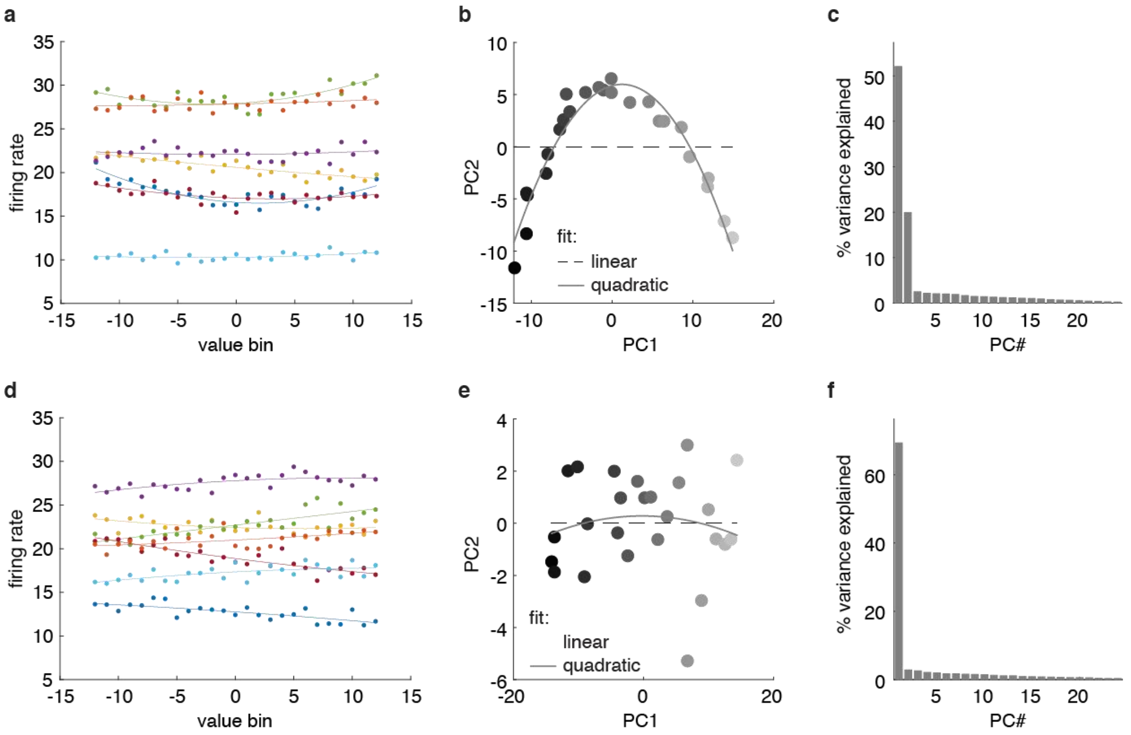

Figure S8. Simulated neural populations that either contained or did not contain curvature, related to Figure 3 and 5. A) *The firing rates of seven example neurons generated with a function that included a quadratic term for curvature (see **Methods**). The lines show best quadratic fit to neurons' responses. **B)** The projection of the simulated neural population (n = 100) onto the first 2 principal components (PCs). Shades of gray = value bins from low (light gray) to high (dark gray). Dotted line = best linear fit. Solid line = best quadratic fit. **C)** Percent variance explained by each PC. **D,E,F)** Same as **(A), (B)** and **(C)**, but for the neuronal responses which were generated as a linear function of the values.*

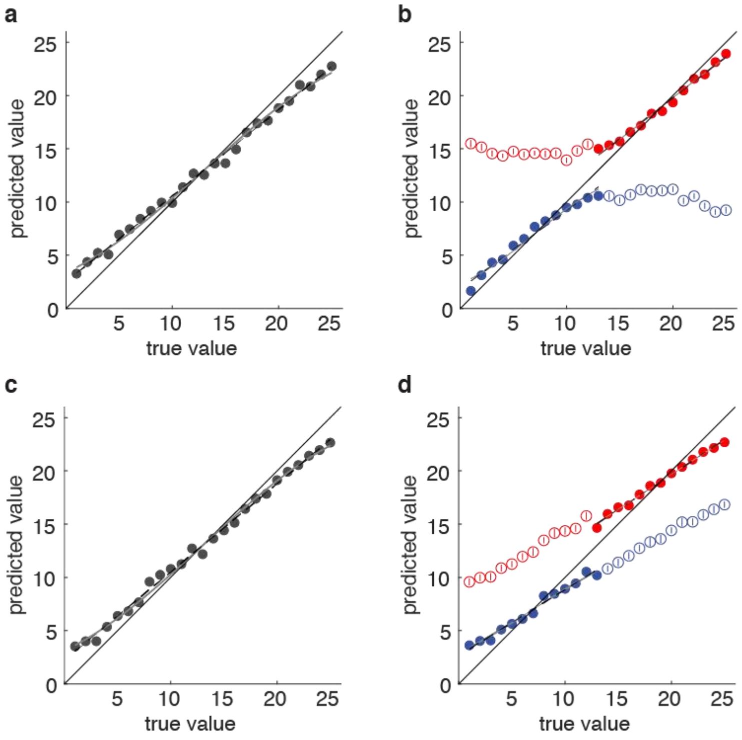

Figure S9. Simulated manifolds reproduce systematic biases in decoding from a curved manifold, related to Figure 4 and 5. A) *After 4B, decoded (predicted) value as a function of true value when decoding from a simulated neural population with curvature.* ***B)*** *After 4E, decoded values for decoders trained only on high (red) or low (blue) values, then used to predict the entire range of values. Filled circles = trained values, open circles = held-out values.* ***C-D)*** *Same as (A-B) for a simulated population without curvature. Error bars indicate +- standard error of the mean across simulated neurons (SEM), n = 100.*

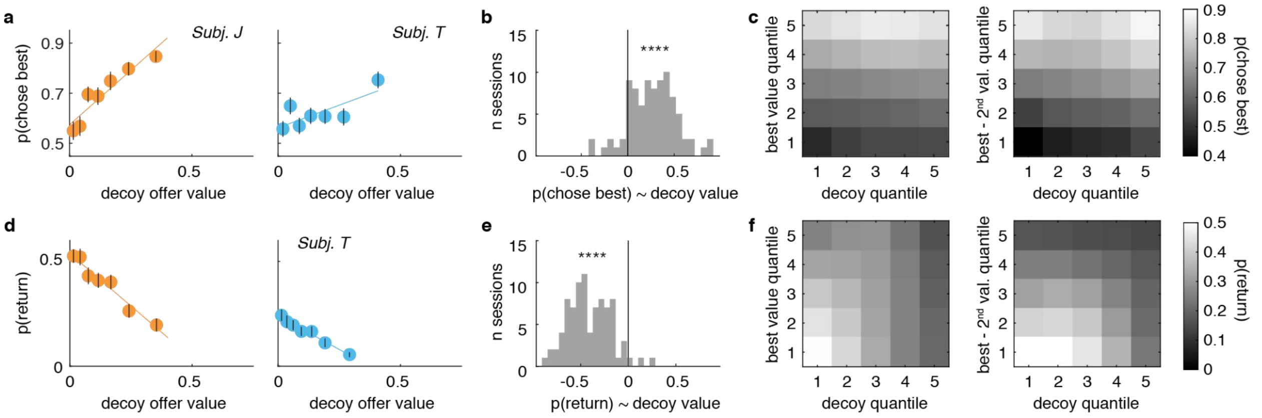

Figure S10. Decoy effect within most confusable trials, related to Figure 5. A) *Probability of choosing the best offer seen as a function of the worst offer seen within trials, in which the best and 2nd best offer values were < 0.2 from each other (n, subject J = 2,937; n, subject T = 2,866). Line = least squares fit.* ***B)*** *Distribution of decoy effects within the probability of choosing the best option in the set across sessions.* ***C)*** *Probability of choosing the best option seen within each trial as a function of both the best value and the decoy value in the set (left) and the difference between the best and 2nd best option, and the decoy value (right).* ***D-F)*** *Same as (**A-C**) but for the probability of return. Error bars indicate +- standard error of the mean across sessions (SEM), subject J: n sessions = 45, subject T: n sessions = 41.****p < 0.001.*

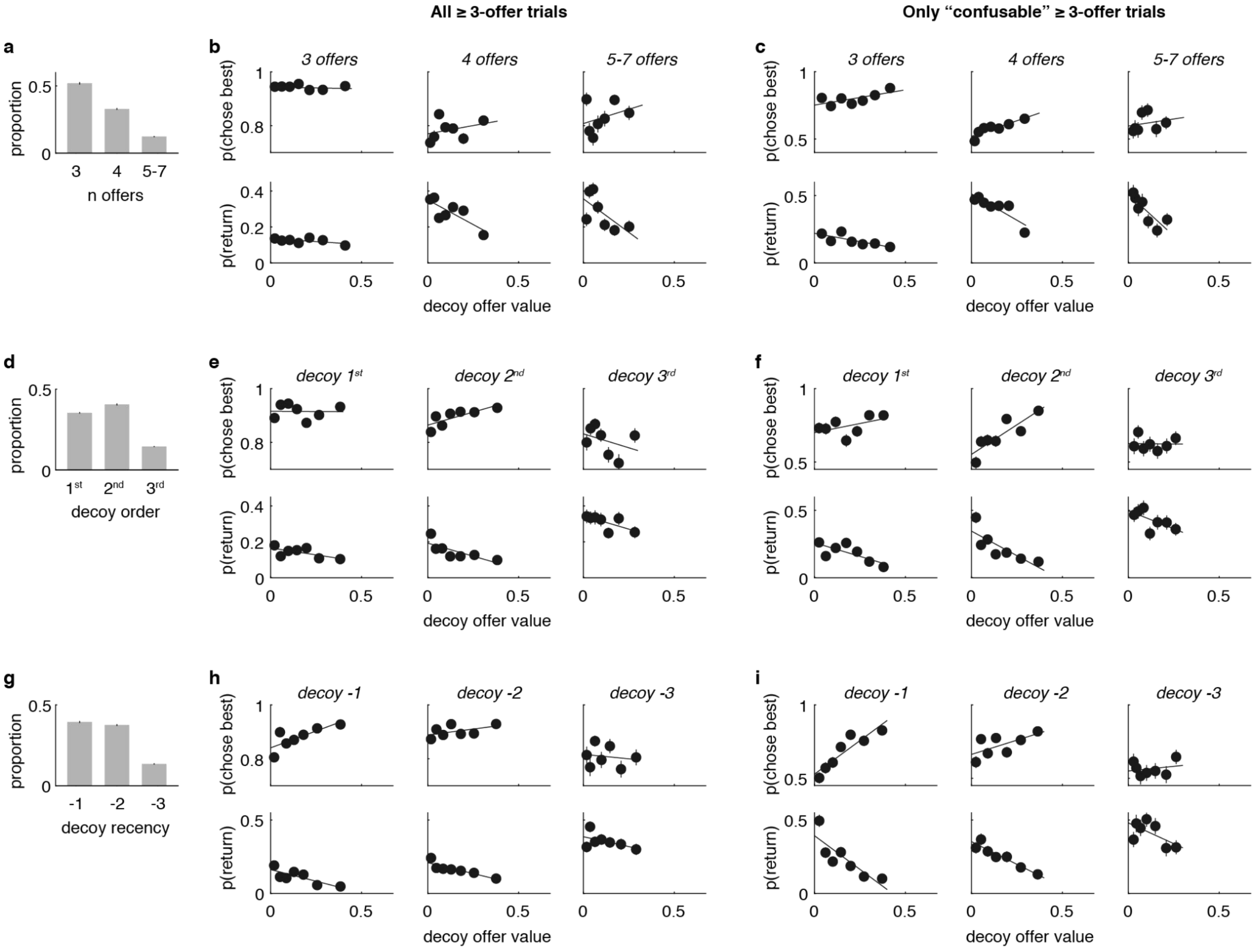

Figure S11. Decoy effect in split for number of offers viewed, decoy order and recency, related to Figure 5. A) *Percent of trials in which subjects revealed 3, 4 or more (5-7) offers.* ***B)*** *Decoy effect for the subset of trials in which subjects revealed 3, 4 or more offers ([GLM that includes number of offers viewed, best value, best-2nd best value, decoy value, and pairwise interactions with decoy value], choice accuracy: mean decoy effect slope = 0.07, range = [-0.22, 0.33], $p < 0.001$, decoy by number of offers interaction, mean slope = 0.07, range = [-0.79, 1.13], $p < 0.0891$; probability of return: mean decoy effect slope = -0.11, range = [-0.48, 0.24], $p < 0.001$; decoy by number of offers interaction, mean slope = -0.40, range = [-1.62, 0.61], $p < 0.001$).* ***C)*** *Same as (**B**), but for the subset of trials in which subjects demonstrated the highest confusion, i.e. trials in which the best and 2nd best offers were within 0.2 value range of each other.* ***D)*** *Percent of trials in which decoy was revealed as first, second or third in the sequence.* ***E)*** *Decoy effect for the subset of trials with the decoy option presented as first, second, third ([GLM that includes decoy order, best value, best-2nd best value, decoy value, and pairwise interactions with decoy value], choice accuracy: mean decoy effect slope = 0.08, range = [-0.54, 0.52], $p < 0.001$, decoy value by decoy order interaction, mean slope = 0.09, range = [-1.01, 1.09], $p < 0.0309$; probability of return: mean decoy effect slope = -0.20, range = [-0.73, 0.46], $p < 0.001$, decoy value by decoy order interaction, mean slope = -0.34, range = [-1.31, 0.52], $p < 0.001$).* ***F)*** *Same as (**E**), but for the trials in which the best and 2nd best offers were within 0.2 value range of each other.* ***G)*** *Percent of trials in which decoy was revealed as -1, -2, or -3 offer before the choice.* ***H)*** *Decoy effect for the subset of trials with the decoy option presented as*

*first, second, third before the last one ([GLM that includes decoy recency, best value, best-2nd best value, decoy value, and pairwise interactions with decoy value], choice accuracy: mean decoy effect slope = 0.13, range = [-0.34, 0.54], p < 0.001, decoy value by decoy recency interaction, mean slope = 0.14, range = [-0.71, 1.04], p < 0.0005; probability of return: mean decoy effect slope = -0.26, range = [-0.86, 0.27], p < 0.001, decoy value by decoy recency interaction, mean slope = -0.27, range = [-1.19, 0.88], p < 0.001). **I)** Same as **(H)**, but for the trials in which the best and 2nd best offers were within 0.2 value range of each other. Line = least squares fit. Error bars in each graph indicate +- standard error of the mean across sessions (SEM), n sessions = 86. These are sometimes smaller than the symbols.*

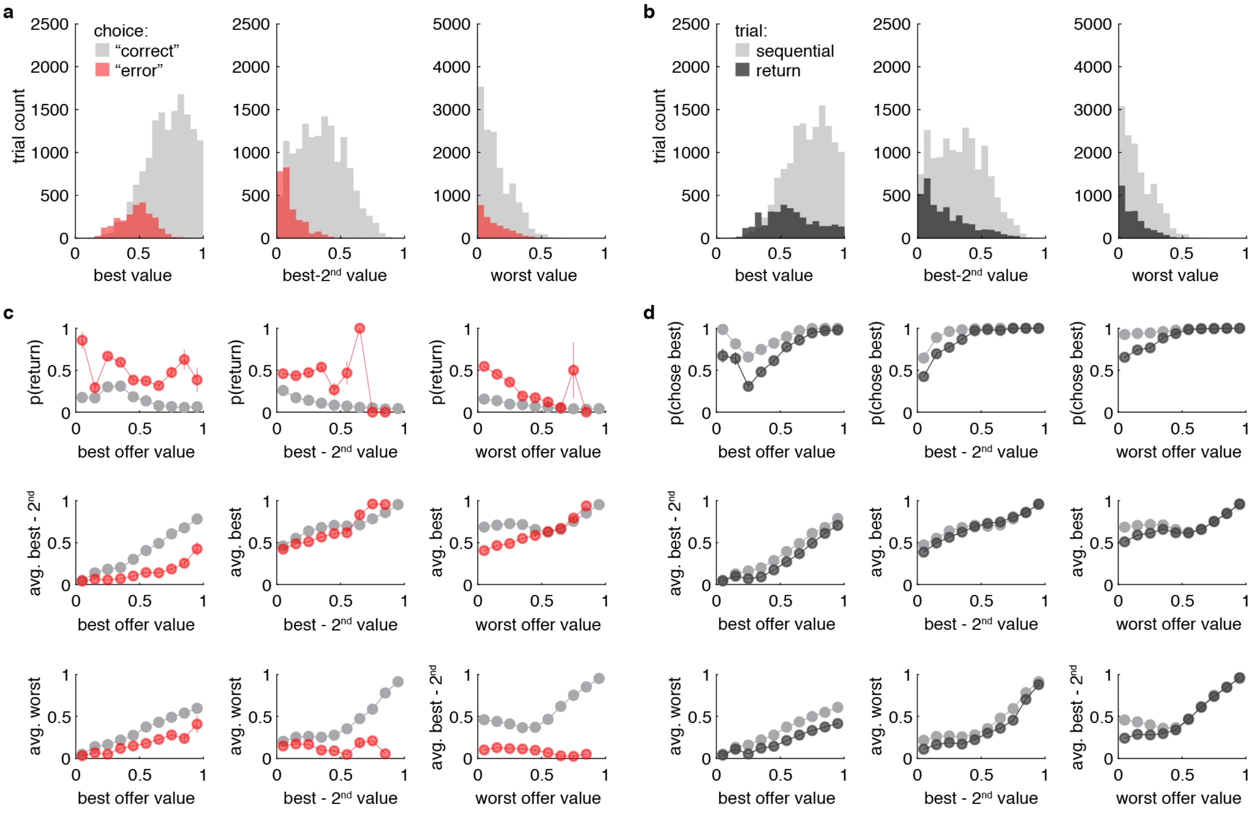

Figure S12. Errors and returns depending on the best, best-2nd best and worst offer values (for trials in which at least 3 offers were revealed), related to Figures 1 and 5. A) *Distribution of trials in which the best offer was chosen (i.e. the choice was "correct"; gray) or some other offer was chosen (i.e. an "error" was made; red), depending on the value of the best, best-2nd best and worst offer revealed in the trial.* ***B)*** *Same as A, but for trials which contained only sequential reveals, (gray) versus those that contained returns (black), depending on the value of the best, best-2nd best and worst offer revealed in the trial.* ***C)*** *Relationships between pairs of reward-related variables, split by "correct" (best offer chosen; gray) and "error" (other offer chosen; red) trials.* ***D)*** *Same as C, but split by purely sequential (gray) versus return (black) trials. Error bars in each graph indicate +- standard error of the mean across sessions (SEM), n sessions = 86. These are sometimes smaller than the symbols.*

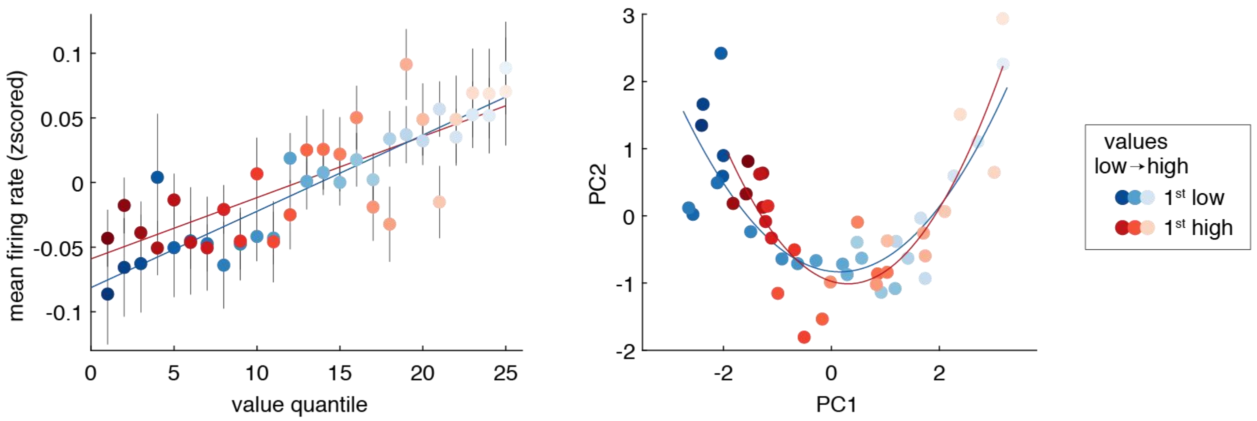

Figure S13. Average neuronal tuning and the curvature of the manifold within trials that started from low and high value offers, related to Figures 2, 3 and 5. *(Left) Average firing rates from all 122 neurons, plotted as a function of value quantile, separately for trials which started from a low (<0.5; blue dots) or a high (>=0.5 value; red dots) value offer. Error bars indicate +- standard error of the mean across neurons (SEM). Lines indicate linear fit. (Right) The projection of the neural population onto the first 2 principal components (PCs), performed separately for trials which started from a low (<0.5; blue dots) or a high (>=0.5 value; red dots) value offer. PCA fit was performed for both pseudopopulations together. Lines indicate quadratic fit. The figure legend is shared by both plots.*

Table S1

Parameter estimates in four-parameter logistic function [choice by value], individual subjects. Related to Figure 1.

parameter	subject J	subject T
slope	16.71	14.31
intercept	0.49	0.57
scale	0.87	0.91
offset	0.03	0.09
R2	0.58	0.57
n	78453	56797

Table S2

Average betas obtained in GLM that included the main effect of the best option value, the difference between the best and second-best option values, the decoy value and the pairwise interactions with the decoy value term, to predict the effects in behavioral responses. T-test from 0 in individual subjects. Related to Figure 5.

	subject J n = 45 sessions				subject T n = 40 sessions			
effect	t(44)	avg. beta	95% CI	p	t(40)	avg. beta	95% CI	p
p(chose best)								
decoy value	10.04	0.30	[0.24, 0.36]	0.001	3.10	0.13	[0.05, 0.21]	0.004
best value	30.72	1.78	[1.67, 1.90]	0.001	33.08	2.21	[2.08, 2.35]	0.001
best-2nd best value	30.02	1.73	[1.61, 1.84]	0.001	34.81	2.07	[1.95, 2.19]	0.001
p(return)								
decoy value	-8.09	-0.45	[-0.56, -0.34]	0.001	-12.21	-0.59	[-0.69, -0.50]	0.001
best value	-15.17	-1.33	[-1.50, -1.15]	0.001	-17.58	-1.71	[-1.90, -1.51]	0.001
best-2nd best value	-11.31	-0.89	[-1.05, -0.73]	0.001	-11.41	-0.83	[-0.98, -0.69]	0.001

Table S3

Average betas obtained in GLM that included the main effect of the best option value, the difference between the best and second-best option values, and the decoy value to predict the effects in behavioral responses. T-test from 0 across sessions in individual subjects. Related to Figure 5.

	subject J n = 45 sessions				subject T n = 41 sessions			
effect	t(44)	avg. beta	95% CI	p	t(40)	avg. beta	95% CI	p
p(chose best)								
decoy value	9.31	0.27	[0.21, 0.33]	0.001	1.67	0.07	[-0.01, 0.15]	0.104
best value	31.05	1.76	[1.64, 1.87]	0.001	33.33	2.18	[2.05, 2.31]	0.001
best-2nd best value	30.07	1.72	[1.60, 1.83]	0.001	34.93	2.06	[1.94, 2.17]	0.001
decoy by best value	24.75	0.24	[0.22, 0.26]	0.001	28.59	0.31	[0.28, 0.33]	0.001
decoy by best-2nd best	18.44	0.34	[0.30, 0.38]	0.001	15.81	0.37	[0.32, 0.41]	0.001
p(return)								
decoy value	-7.93	-0.39	[-0.49, -0.29]	0.001	-11.88	-0.51	[-0.60, -0.43]	0.001
best value	-15.11	-1.30	[-1.47, -1.12]	0.001	-17.48	-1.66	[-1.86, -1.47]	0.001
best-2nd best value	-11.28	-0.89	[-1.04, -0.73]	0.001	-11.41	-0.83	[-0.98, -0.68]	0.001
decoy by best value	-9.00	-0.34	[-0.42, -0.26]	0.001	-15.79	-0.52	[-0.59, -0.45]	0.001
decoy by best-2nd best	-10.14	-0.16	[-0.19, -0.13]	0.001	-13.10	-0.19	[-0.22, -0.16]	0.001
