# Supplementary material for: Irrational choices via a curvilinear representational geometry for value
Source: Nat Commun. 2024 Jul 30;15:6424. doi: 10.1038/s41467-024-49568-4 (PMC11289086; doi:10.1038/s41467-024-49568-4)
Supplement: Supplementary file 3 — Reporting Summary [file 41467_2024_49568_MOESM3_ESM.pdf]

nature portfolio

Corresponding author(s): R. Becket Ebitz

Last updated by author(s): May 3, 2024

Reporting Summary

Nature Portfolio wishes to improve the reproducibility of the work that we publish. This form provides structure for consistency and transparency in reporting. For further information on Nature Portfolio policies, see our Editorial Policies and the Editorial Policy Checklist.

Statistics

For all statistical analyses, confirm that the following items are present in the figure legend, table legend, main text, or Methods section.

n/a	Confirmed	
☐	☒	The exact sample size (*n*) for each experimental group/condition, given as a discrete number and unit of measurement
☐	☒	A statement on whether measurements were taken from distinct samples or whether the same sample was measured repeatedly
☐	☒	The statistical test(s) used AND whether they are one- or two-sided<br>*Only common tests should be described solely by name; describe more complex techniques in the Methods section.*
☐	☒	A description of all covariates tested
☐	☒	A description of any assumptions or corrections, such as tests of normality and adjustment for multiple comparisons
☐	☒	A full description of the statistical parameters including central tendency (e.g. means) or other basic estimates (e.g. regression coefficient) AND variation (e.g. standard deviation) or associated estimates of uncertainty (e.g. confidence intervals)
☐	☒	For null hypothesis testing, the test statistic (e.g. *F*, *t*, *r*) with confidence intervals, effect sizes, degrees of freedom and *P* value noted<br>*Give P values as exact values whenever suitable.*
☒	☐	For Bayesian analysis, information on the choice of priors and Markov chain Monte Carlo settings
☐	☒	For hierarchical and complex designs, identification of the appropriate level for tests and full reporting of outcomes
☐	☒	Estimates of effect sizes (e.g. Cohen's *d*, Pearson's *r*), indicating how they were calculated

Our web collection on statistics for biologists contains articles on many of the points above.

Software and code

Policy information about availability of computer code

Data collection	Brainsight system (Rogue Research Inc.) was used for verifying position of the implants<br>Matlab (Mathworks) with Psychtoolbox and Eyelink Toolbox controlled the task presentation<br>Plexon system (Plexon, Inc.) was used for isolating individual action potentials
Data analysis	all analyses were done with custom Matlab code, available at https://osf.io/8mkrd/

For manuscripts utilizing custom algorithms or software that are central to the research but not yet described in published literature, software must be made available to editors and reviewers. We strongly encourage code deposition in a community repository (e.g. GitHub). See the Nature Portfolio guidelines for submitting code & software for further information.

Data

Policy information about availability of data

All manuscripts must include a data availability statement. This statement should provide the following information, where applicable:

- Accession codes, unique identifiers, or web links for publicly available datasets
- A description of any restrictions on data availability
- For clinical datasets or third party data, please ensure that the statement adheres to our policy

All the data used in this study are available in the OSF under accession code https://osf.io/8mkrd/.

Research involving human participants, their data, or biological material

Policy information about studies with human participants or human data. See also policy information about sex, gender (identity/presentation), and sexual orientation and race, ethnicity and racism.

Reporting on sex and gender	n/a
Reporting on race, ethnicity, or other socially relevant groupings	n/a
Population characteristics	n/a
Recruitment	n/a
Ethics oversight	n/a

Note that full information on the approval of the study protocol must also be provided in the manuscript.

Field-specific reporting

Please select the one below that is the best fit for your research. If you are not sure, read the appropriate sections before making your selection.

☒ Life sciences ☐ Behavioural & social sciences ☐ Ecological, evolutionary & environmental sciences

For a reference copy of the document with all sections, see nature.com/documents/nr-reporting-summary-flat.pdf

Life sciences study design

All studies must disclose on these points even when the disclosure is negative.

Sample size	Data were collected in two rhesus macaque monkeys, as we have an ethical mandate to minimize the number of animals that we use. Number of cells recorded (122 in total) has been proved sufficient in a number of previous neural population analyses (e.g. Okazawa et al. 2021).
Data exclusions	One out of 122 cells had to be excluded in state space analyses due to lack of spiking activity in the analyzed time window.
Replication	The results have passed internal replication--all the effects observed in one monkey were also observed in the other monkey (effects tested independently in two subjects.
Randomization	There was no group comparison in the study.
Blinding	Blinding was not relevant to this study--it did not compare groups.

Reporting for specific materials, systems and methods

We require information from authors about some types of materials, experimental systems and methods used in many studies. Here, indicate whether each material, system or method listed is relevant to your study. If you are not sure if a list item applies to your research, read the appropriate section before selecting a response.

Materials & experimental systems

n/a	Involved in the study	
☒	☐	Antibodies
☒	☐	Eukaryotic cell lines
☒	☐	Palaeontology and archaeology
☐	☒	Animals and other organisms
☒	☐	Clinical data
☒	☐	Dual use research of concern
☒	☐	Plants

Methods

n/a	Involved in the study	
☒	☐	ChIP-seq
☒	☐	Flow cytometry
☒	☐	MRI-based neuroimaging

Animals and other research organisms

Policy information about studies involving animals; ARRIVE guidelines recommended for reporting animal research, and Sex and Gender in Research

Laboratory animals	Subjects were two male rhesus macaques (Macaca mulatta): subject J age 10 years; subject T age 5 years.
Wild animals	The study did not involve wild animals.
Reporting on sex	Sex was not considered in study design because we aimed to minimize the number of primate subjects tested, and both subjects were male.
Field-collected samples	The study did not involve samples collected from the field.
Ethics oversight	All procedures were designed and conducted in compliance with the Public Health Service's Guide for the Care and Use of Animals and approved by the University Committee on Animal Resources at the University of Rochester.

Note that full information on the approval of the study protocol must also be provided in the manuscript.

Plants

Seed stocks	n/a
Novel plant genotypes	n/a
Authentication	n/a
